# Dietary intake of pantothenic acid is associated with cerebral amyloid burden in patients with cognitive impairment

**DOI:** 10.29219/fnr.v62.1415

**Published:** 2018-12-10

**Authors:** Jae-Ho Lee, Soo-Yeon Ahn, Hyon Ah Lee, Kyoung Sook Won, Hyuk Won Chang, Jungsu S. Oh, Hae Won Kim

**Affiliations:** 1 Department of Anatomy, Keimyung University Dongsan Medical Center, Daegu, Republic of Korea; 2 Department of Neurology, Keimyung University Dongsan Medical Center, Daegu, Republic of Korea; 3 Department of Nuclear Medicine, Keimyung University Dongsan Medical Center, Daegu, Republic of Korea; 4 Department of Radiology, Semyung Radiology Clinic, Gumi, Republic of Korea; 5 Department of Nuclear Medicine, Asan Medical Center, University of Ulsan College of Medicine, Seoul, Republic of Korea

**Keywords:** Alzheimer’s disease, mild cognitive impairment, pantothenic acid, subjective cognitive impairment, vitamin, diet

## Abstract

Alzheimer’s disease (AD) is a neurodegenerative disease characterized by the deposition of amyloid-β peptide (Aβ) in diffuse and neuritic plaques. Previous research has suggested that certain vitamins may prevent this process. In the present study, we evaluated the relationship between vitamin intake and cerebral Aβ burden in patients with cognitive impairment. This study included 19 patients with subjective cognitive impairment and 30 patients with mild cognitive impairment. All patients underwent brain MRI and ^18^F-florbetaben positron emission tomography. The Food Frequency Questionnaire was used to evaluate dietary intake of the 15 vitamins. Intake of vitamin B6 (*p* = 0.027), vitamin K (*p* = 0.042), vitamin A (*p* = 0.063), riboflavin (*p* = 0.063), β-carotene (*p* = 0.081), pantothenic acid (*p* = 0.092), and niacin (*p* = 0.097) was higher in the Aβ-positive group than in the Aβ-negative group. Multivariate linear regression analysis revealed that pantothenic acid intake was an independent determinant of cerebral Aβ burden (β = 0.287, *p* = 0.029). No significant correlations were observed between cerebral Aβ burden and the intake of other vitamins. Our findings demonstrated that pantothenic acid intake may be associated with increased cerebral Aβ burden in patients with cognitive impairment. These results may offer insight into potential strategies for AD prevention.

The prevalence and economic costs of Alzheimer’s disease (AD) have continued to increase along with increases in the number of older adults in the population (1). Although the diagnosis of AD is primarily based on clinical symptoms, recent advances in genetics and neuroimaging have indicated that these modalities may aid in the diagnosis of AD (2–4). Additional research has suggested that positron emission tomography (PET) with ^18^F-florbetaben (^18^F-FBB) can be used to identify cerebral amyloid-β (Aβ) pathology in patients undergoing assessments for AD (5, 6). They showed a sensitivity of 80% and a specificity of 91% of PET scan for the diagnosis of Alzheimer’sdisease (6).

Although the etiology of the disease remains to be fully established, evidence suggests that the Aβ peptide plays an important role in the pathogenesis of AD (5–7). In addition, several risk factors for AD have been identified, including age, certain genetic alleles, and specific nutritional characteristics (8–12). Accumulating evidence indicates that diet – one of the most important modifiable lifestyle factors – may play a role in preventing or delaying cognitive decline and AD (13–16). Epidemiological studies have reported that low intake of vitamins increases the risk of AD, and that several vitamins may be associated with the pathological processes of AD (17–20). However, whether vitamin intake influences the accumulation of cerebral Aβ remains to be investigated.

Previous studies have established the validity of reliability on the Food Frequency Questionnaire (FFQ), which was developed to assess the association between chronic diseases and diet in Korean populations (21, 22). In the present study, we aimed to evaluate the association between vitamin intake and cerebral Aβ burden in patients with cognitive impairment using ^18^F-FBB PET and the FFQ. We hope that the results of our study will improve our understanding of AD pathogenesis, which may aid in the treatment and prevention of AD.

## Materials and methods

### Study population

The present prospective study included consecutive patients (age range: 50–90 years) who had visited the memory clinic at Keimyung University Dongsan Medical Center (Daegu, Korea) for the evaluation of cognitive function between June 2015 and January 2017. All patients underwent standard clinical and neuropsychological evaluations. All patients were divided into syndromal categories based on the 2018 National Institute on Aging-Alzheimer’s Association Research Framework: subjective cognitive impairment (SCI) and mild cognitive impairment (MCI) (23). The Mini-Mental State Examination (MMSE), Digit Span Memory Test, Korean-Boston Naming Test (K-BNT), Rey-Osterrieth Complex Figure Test, and Recognition Trial (RCFT) were used to assess cognitive function (24). SCI was defined as subjective memory disorder wherein patients report worsening of their thinking abilities, including memory, but the decline cannot be verified by the tests. MCI was defined as a condition in which subjects had mild but measurable changes in thinking abilities that are noticeable to the person affected and to family members and friends, but do not affect the individual’s ability to carry out everyday activities (2). All participants underwent FFQ, brain magnetic resonance imaging, and ^18^F-FBB PET within 4 weeks of visiting the clinic. Patients with AD or an MMSE score <20 were excluded to ensure the reliability of the FFQ. Patients with conditions that could affect cognition (e.g. vascular dementia, a history of psychiatric episodes or substance abuse, or a previous diagnosis of dementia) were also excluded. And patients with other neurodegenerative diseases (e.g. Parkinson’s disease); inflammatory brain diseases (multiple sclerosis); medications history such as sedatives, tranquilizers, and anticholinergics; and cancers were also excluded. The study was approved by the Institutional Review Board of Dongsan Medical Center, and written informed consent was obtained from all participants or caregivers.

### Dietary assessment

The categories listed in the FFQ were based on questions from the 2005 Korean Health and Nutrition Survey. A well-trained dietary interviewer used the FFQ to record participants’ typical dietary intake. The questionnaire consisted of a list of foods with standard serving sizes, for which patients were requested to select from among nine frequency categories: three times daily, twice daily, once daily, five or six times weekly, three or four times weekly, once or twice weekly, two or three times monthly, once monthly, and never or seldom. Portion sizes were classified into three categories (i.e. small, medium, and large) based on the patient’s intake in relation to an appropriately defined unit (e.g. cup or bowl). Dietary intake of nutrients and food groups was assessed using computerized assessment software (CAN-Pro 4.0, Korean Nutrition Society, Seoul, Korea).

### Amyloid PET

A PET/CT system (Biograph mCT-64, Siemens Healthcare, Knoxville, TN) was used to acquire three-dimensional ^18^F-FBB PET images 90–100 min after the intravenous injection of ^18^F-FBB (300 MBq). Nonenhanced, low-dose CT was performed for attenuation correction and localization. A light, foam-rubber holder was used for fixation of the head. The PET images were subjected to iterative reconstruction using ordered subset expectation maximization. Attenuation correction of PET images was performed using attenuation data from the CT images.

Quantitative analyses were conducted on volumes of interest (VOI) using PMOD software (PMOD Technologies Ltd., Zurich, Switzerland), as previously described (5). Image processing was performed using SPM12 (Wellcome Department of Imaging Neuroscience, Institute of Neurology, University College London) implemented in MATLAB 2013a (MathWorks Inc., MA, USA) and MRIcro version 1.37 (Chris Rorden, Columbia, SC, USA, www.mricro.com).

Each MRI and PET image was co-registered using a standard mutual information algorithm and spatially normalized. An automated anatomical labeling template was subsequently applied for standardized sampling of count densities in VOIs. VOIs were individually defined in the bilateral frontal, temporal, and parietal cortices; anterior and posterior cingulate; and cerebellar cortex. Standardized uptake values were obtained from the regional VOIs, and regional standardized uptake value ratios (SUVRs) were calculated by dividing the standardized uptake values for the different target regions by that for the reference region (i.e. cerebellar cortex). A composite SUVR was calculated as the mean of the values for the frontal, parietal, lateral, temporal, and occipital cortices as well as the anterior and posterior cingulate, as previously described (6). Regional and composite SUVRs were used to evaluate the relationship between vitamin intake and cerebral Aβ burden. Patients with a composite SUVR ≥ 1.5, which is considered indicative of an abnormally high cerebral Aβ burden, were considered positive for Aβ (Aβ-positive group), while those with a composite SUVR < 1.5 were considered negative for Aβ (Aβ-negative group) (6). We then compared differences in vitamin intake between the Aβ-positive and Aβ-negative groups. This analysis was performed in blinded manner.

### Statistical analyses

All statistical analyses were performed using IBM SPSS statistics for Windows, V.20 (IBM Corp). Mann–Whitney U tests, Kruskal–Wallis tests, and simple correlation analyses were used to analyze associations between variables. We also assessed the relationship between vitamin intake, MMSE score, and SUVR (cerebral Aβ burden) using Pearson’s correlation coefficients and multiple linear regression analyses adjusted for age, sex, and body mass index (BMI). *APOE*4 as a covariate in the primary model was excluded to avoid variance inflation given its high correlation with cerebral Aβ burden (25, 26). A *P-*value of < 0.05 was considered statistically significant. Data for all study variables are expressed as means ± standard deviations.

## Results

A total of 49 patients with cognitive impairment were enrolled in the present study. Among them, 19 patients were clinically diagnosed with SCI, while the remaining 30 were diagnosed with MCI. The demographic, clinical, and biochemical characteristics of the SCI and MCI groups are shown in Table 1. MMSE scores were significantly lower in patients with MCI than in those with SCI (*p* < 0.001). Increased cerebral Aβ burden was observed in 10.5% (2/19) of patients with SCI and 23.3% (7/30) of patients with MCI, although there was no significant difference between the groups (*p* = 0.247). A negative correlation between cerebral Aβ burden and MMSE score was shown (*r* = −0.395, *p* = 0.007).

**Table 1:** Characteristics of patients^a^

	SCI	MCI	*P value*
	(n = 19)	(n = 30)	
Age, y	62.7 (5.5)	64.3 (9.7)	0.540
Female, %	46.4	53.6	0.763
BMI	24.3 (4.4)	23.6 (2.1)	0.479
Education, y	14.33 (3.5)	12.8 (5.6)	0.310
*APOE*4+, %	21.8	24.3	0.610
SUVR for Aβ	1.26 (0.12)	1.35 (0.23)	0.140
MMSE	28.9 (1.2)	25.7 (2.6)	< 0.001
Digit span	11.7 (2.3)	10.0 (2.2)	< 0.001
K-BNT	51.8 (3.9)	46.3 (9.4)	< 0.001
RCFT	33.0 (2.4)	30.6 (5.3)	< 0.001

a All values are means (SD). K-BNT: Korean-Boston Naming Test; RCFT: Rey-Osterrieth Complex Figure Test and Recognition Trial.

Vitamin intake for the Aβ-negative and Aβ-positive groups is presented in Table 2. Intake of vitamin K (*p* = 0.042) and vitamin B6 (*p* = 0.027) was significantly higher in the Aβ-positive group than in the Aβ-negative group. Similar tendencies were observed for vitamin A (*p* = 0.063), β-carotene (*p* = 0.081), riboflavin (*p* = 0.063), niacin (*p* = 0.097), and pantothenic acid (*p* = 0.092), although these results did not reach statistical significance. When stratifying patients into SCI and MCI, a correlation between cerebral Aβ burden and intake of pantothenic acid was found not in SCI (*r* = −0.126, *p* = 0.618) but in MCI (*r* = 0.494, *p* = 0.008).

**Table 2:** Comparisons of vitamin intake between Aβ negative and Aβ positive groups^a^

	Aβ negative (n = 37)	Aβ positive (n = 9)	*P value*
Vitamin A (ug)	1392.2 (1161.0)	1966.4 (1143.8)	.063
Retinol (ug)	182.3 (167.8)	189.9 (79.2)	.207
β-carotene (ug)	6949.5 (5962.8)	10144.4 (6721.2)	.081
Vitamin D (ug)	2.8 (2.4)	2.6 (1.7)	1.00
Vitamin E (mg)	6.9 (8.4)	7.5 (2.9)	.109
Vitamin K (ug)	36.5 (30.0)	54.7 (29.7)	**.042**
Vitamin C (mg)	267.1 (272.3)	333.4 (172.3)	.116
Thyamine (mg)	2.8 (2.7)	3.4 (1.5)	.097
Riboflavin (mg)	2.5 (2.2)	3.0 (1.0)	.063
Niacin (mg)	19.9 (16.6)	24.6 (9.9)	.097
Vitamin B6 (mg)	0.5 (0.5)	0.6 (0.1)	**.027**
Folic acid (ug)	223.8 (197.6)	249.2 (121.8)	.286
Vitamin B12 (ug)	3.0 (4.4)	3.3 (2.3)	.273
Pantothenic acid (mg)	1.2 (0.9)	1.6 (0.9)	.092
Biotin (ug)	6.5 (3.8)	7.9 (5.6)	.605

a All values are means (SD).

**Fig. 1:**
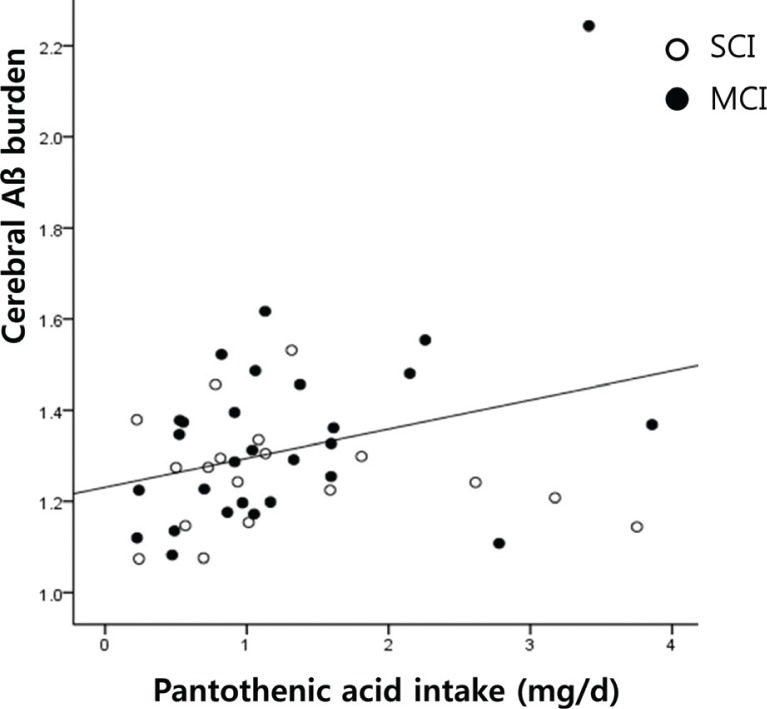
Positive correlation between pantothenic acid intake and cerebral β-amyloid burden in SCI and MCI.

Pearson’s correlation analysis revealed that the intake of pantothenic acid was positively correlated with composite SUVR(*r* = 0.303, *p* = 0.041; Fig.1). When stratified by SUVR, this correlation was found not in Aβ-negative group (*r* = 0.034, *p* = 0.844) but in Aβ-positive group (*r* = 0.789, *p* = 0.012). No additional associations were observed between vitamin intake and composite SUVR. The results of the multiple linear regression analysis adjusted for age, sex, and BMI are presented in Table 3. Intake of pantothenic acid was identified as an independent determinant of composite SUVR(*R*^2^ = 0.289, *p* = 0.029). No such independent relationships were observed between composite SUVR and other vitamins.

**Table 3:** The relationships of cerebral β-amyloid burden with vitamin intake

	Adjusted *R*^2^	Standardized *β*	*P value*
Vitamin A	—	.115	.398
Retinol	—	.073	.593
β-carotene	—	.103	.450
Vitamin D	—	.100	.456
Vitamin E	—	.079	.564
Vitamin K	—	.140	.306
Vitamin C	—	−.010	.942
Thyamine	—	.104	.443
Riboflavin	—	.103	.451
Niacin	—	.108	.429
Vitamin B6	—	.133	.333
Folic acid	—	.126	.362
Vitamin B12	—	.132	.326
Pantothenic acid	—	.287	**.029**
Biotin	—	.096	.487

We then divided patients into two groups based on median pantothenic acid intake (1.27 mg), following which we compared regional cerebral Aβ burden between patients with high and low pantothenic acid intake (Table 4). In all cerebral regions, regional SUVR was significantly higher in the high-intake group than in the low-intake group. Multivariate analyses adjusted for age, sex, and BMI revealed that regional SUVR in the frontal, parietal, and temporal cortices was associated with pantothenic acid intake (Table 5). Although a similar tendency was observed in the cingulate cortices, this result did not reach statistical significance (right, *p* = 0.073 and left, *p* = 0.063).

**Table 4:** Comparison of regional SUVR between pantothenic acid low-intake and high-intake groups^a^

Region	Side	pantothenic acid		*P value*
		Low-intake (n = 19)	High-intake (n = 30)	
Frontal lobe	Rt	1.15 (0.11)	1.29 (0.21)	**0.01**
	Lt	1.17 (0.12)	1.32 (0.21)	**0.01**
Parietal lobe	Rt	1.17 (0.14)	1.29 (0.29)	**0.06**
	Lt	1.25 (0.13)	1.39 (0.32)	**0.04**
Temporal lobe	Rt	1.22 (0.12)	1.33 (0.19)	**0.02**
	Lt	1.31 (0.12)	1.43 (0.24)	**0.03**
Cingulate	Rt	1.56 (0.16)	1.72 (0.27)	**0.02**
	Lt	1.44 (0.17)	1.60 (0.35)	**0.05**

a All values are mean Fazekas rating.

**Table 5:** The relationships of regional cerebral β-amyloid burden with intake of pantothenic acid

Region	Side	Adjusted *R*^2^	Standardized *β*	*P value*
Frontal lobe	Rt	0.279	0.272	**0.039**
	Lt	0.305	0.321	**0.014**
Parietal lobe	Rt	0.194	0.309	**0.030**
	Lt	0.266	0.267	**0.044**
Temporal lobe	Rt	0.297	0.295	**0.024**
	Lt	0.396	0.339	**0.006**
Cingulate	Rt	—	0.248	0.073
	Lt	—	0.242	0.063

## Discussion

In the present study, we aimed to evaluate the relationship between vitamin intake and cerebral Aβ burden in patients with cognitive impairment. Some of SCI population and patients with MCI had Aβ positive result. Although subjects with Aβ positive are not cognitively impaired, these would be changed into dementia within 20 years. Our results indicated that cerebral Aβ burden was positively associated with dietary intake of pantothenic acid in patients with MCI and SCI. Interestingly, subjects with Aβ positive showed this correlation with more significance. Furthermore, higher intake of pantothenic acid from food sources was linearly associated with cerebral Aβ burden in various brain regions, even after controlling for multiple covariates.

In general, humans require adequate amounts of four fat-soluble vitamins (A, D, E, K) and nine water-soluble vitamins, which comprise vitamin C and the eight B vitamins: thiamine (B_1_), riboflavin (B_2_), niacin (B_3_), pantothenic acid (B_5_), vitamin B_6_, vitamin B_7,_ folate (B_9_), and vitamin B_12_ (17, 18). Pantothenic acid (B_5_) is a substrate for the synthesis of the ubiquitous coenzyme A (CoA) (27). Beyond its role in oxidative metabolism, CoA contributes to the structure and function of brain cells via its involvement in the synthesis of cholesterol, amino acids, phospholipids, and fatty acids (28, 29). Notably, pantothenic acid is also involved in the synthesis of multiple neurotransmitters and steroid hormones via pathways involving CoA (28).

Supplementation of several vitamins such as folate, vitamin B_12_, and vitamin B_6_ has been recommended for the prevention of cognitive decline (14–19). In contrast, our results indicate that increased pantothenic acid intake may increase cerebral Aβ burden. And over intakes of Vitamin K and Vitamin B6 tended to be risk factor for Aβ burden uptake. Vitamin K and Vitamin B6 tended to be risk for Aβ burden uptake. However, multivariate analysis showed that only pantothenic acid is a strong factor to increase cerebral Aβ burden.

Major food sources of pantothenic acid include chicken, beef, potatoes, oat cereals, tomatoes, eggs, broccoli, and whole grains (29). Previous study showed an average pantothenic acid intake (4.5 mg/day for men and 4.0 mg/day for women), which is higher than our result (27). As previously stated, total intake of pantothenic acid may be low because of its widespread occurrence in food. Although these foods are well-known sources of neuroprotection (30, 31), excessive intake may induce unexpected effects on CoA pathways, proteins, and carbohydrate synthesis. Pantothenic acid, a precursor to CoA, plays an indispensable role in carboxylic acid and fatty acid metabolism in various organisms (32). Therefore, increased CoA activity via high pantothenic acid intake may induce inappropriate consumption of biological defense resources such as pro-inflammatory cytokines, adhesion molecules, and acute response proteins associated with inflammation. However, further studies are required to verify this hypothesis.

The present study possesses several limitations of note. First, the FFQ is limited in assessing absolute intake because it is based on a finite list of food items, categories, and intake levels. And the intake of other compounds and health supplements also influence the cerebral Aβ burden though they were included in FFQ. Therefore, evaluation using the FFQ should be followed by analysis of nutrient levels in the blood. In addition, because all study participants resided in South Korea, we were unable to examine the influence of regional differences in environment, socioeconomic status, and health-related habits. Thus, further prospective studies involving various populations are required. Despite these limitations, the present study is advantageous in that a novel cross-sectional approach was used to investigate the association between dietary intake of pantothenic acid and cerebral Aβ burden.

## Conclusion

The present study is the first to demonstrate a positive association between cerebral Aβ burden and dietary intake of pantothenic acid in patients with cognitive impairment using PET and the FFQ. Notably, this association was observed in most cerebral regions and following multivariate analysis, suggesting that pantothenic acid intake influences cerebral Aβ burden. Further studies are required to confirm our findings and to elucidate the molecular mechanisms underlying this association.
